# LncRNA OIP5-AS1 Knockdown Targets miR-183-5p/GLUL Axis and Inhibits Cell Proliferation, Migration and Metastasis in Nasopharyngeal Carcinoma

**DOI:** 10.3389/fonc.2022.921929

**Published:** 2022-06-08

**Authors:** Shuo Li, Mingxing Tang, Nan Zen, Junyi Liang, Xiao Xing, Danglin Huang, Fei Liu, Xiaomeng Zhang

**Affiliations:** ^1^ Department of Otolaryngology, The 6th Affiliated Hospital of Shenzhen University Health Science Center, Shenzhen, China; ^2^ Department of Otolaryngology, Huazhong University of Science and Technology Union Shenzhen Hospital, Shenzhen, China; ^3^ Department of Otolaryngology, Affiliated Shenzhen Sixth Hospital of Guangdong Medical University, Shenzhen, China; ^4^ Department of Otorhinolaryngology, Union Hospital, Tongji Medical College, Huazhong University of Science and Technology, Wuhan, China

**Keywords:** nasopharyngeal carcinoma, OIP5-AS1, miR-183-5p, GLUL, viability

## Abstract

Nasopharyngeal carcinoma (NPC) is often associated with the infection of Epstein-Barr virus in nasopharynx and is mainly happened in South China and Southeast Asia. Recently, noncoding RNAs have been reported to regulate NPC carcinogenesis. LncRNA OIP5-AS1 participates in tumorigenesis and progression; however, the inherent mechanism of OIP5-AS1-mediated progression of NPC is unclear. In the current study, we aimed to explore the role of OIP5-AS1 in NPC progression. We measured the cell viability, apoptosis, migration, and invasion in NPC cells after OIP5-AS1 modulation. Moreover, we determined whether OIP5-AS1 exerts its oncogenic functions *via* sponging miR-183-5p in NPC. Furthermore, we determined whether glutamate ammonia ligase (GLUL) was a downstream target of miR-183-5p. We found that OIP5-AS1 downregulation inhibited the viability, migration and invasion of NPC *via* targeting miR-183-5p. We also identified that GLUL might be a potential downstream target of miR-183-5p in NPC cells. Mechanistically, OIP5-AS1 promotes cell motility *via* regulating miR-183-5p and GLUL in NPC cells. We concluded that OIP5-AS1 performed its biological functions *via* targeting miR-183-5p and GLUL in NPC cells.

## Introduction

Nasopharyngeal carcinoma (NPC) is often associated with the infection of Epstein-Barr virus in nasopharynx (1). This disease generally has ethnic and geographic features, such as in South China and Southeast Asia (2). Many factors have been believed to contribute to NPC development, including EBV infection, genetic susceptibility, dietary habits, smoking, epigenetic alterations. The current therapy for NPC patients includes chemotherapy and radiotherapy (3–5). NPC patients often have recurrence, leading to poor overall survival (6). Therefore, it is important to determine the molecular mechanism of NPC development and progression, which is still not thoroughly elucidated.

Recently, noncoding RNAs have been reported to regulate NPC carcinogenesis (7, 8). Several studies have revealed that lncRNA OIP5-AS1 participates in tumorigenesis and progression (9–11). Higher expression of OIP5-AS1 could be associated with an advanced stage and a poor survival in multiple cancer types (12). Zhang et al. reported that depletion of OIP5-AS1 suppressed cell proliferation, EMT and metastasis *via* upregulation of miR-186a-5p and inhibition of ZEB1 in hepatoblastoma cells (13). Wang et al. found that OIP5-AS1 increased cell proliferation *via* sponging miR-378a-3p in lung cancer (14). Similarly, OIP5-AS1 enhanced lung cancer stemness *via* promotion of Oct4 mRNA stability (15). One group identified that OIP5-AS1 facilitated cell proliferation and invasive activity *via* interacting with miR-143-3p and increasing integrin alpha6 expression in cervical cancer (16). Similarly, OIP5-AS1 promoted viability, migration and invasion of cervical cancer cells *via* binding with miR-143-3p and upregulating SMAD3 expression in cervical cancer cells (17). OIP5-AS1 increased invasion, migration and EMT *via* targeting miR-147a and IGF1R in cervical cancer (18). OIP5-AS1 inhibited miR-92a and increased cell growth and metastasis *via* upregulation of ITGA6 in ovarian cancer (19). OIP5-AS1 interacted with miR-34a and increased the PD-L1 expression in NSCLC cells (20). Depletion of OIP5-AS1 retarded cell growth, migration and stimulated apoptosis *via* targeting miR-129-5p and SOX2 in breast cancer (21). Knockdown of OIP5-AS1 expression attenuated cell viability, triggered cell cycle arrest and activated apoptosis in bladder cancer (22).

Multiple researches demonstrated that OIP5-AS1 exerts anti-tumor functions in various types of cancers. For example, one study showed that OIP5-AS1 reduced clonogenic survival and induced apoptosis in colorectal cancer cells after irradiation *via* targeting miR-369-3p and DYRK1A, suggesting that OIP5-AS1 enhances radio-sensitivity in colorectal cancer (23). One study revealed that OIP5-AS1 enhanced NPC progression *via* sponging miR-203 (24). The inherent mechanism of OIP5-AS1-mediated progression of NPC is unclear. In the current study, we investigated the role of OIP5-AS1 in NPC progression. To achieve this goal, we measured the cell viability, apoptosis, migration, and invasion in NPC cells after OIP5-AS1 modulation. Moreover, we determined whether OIP5-AS1 exerts its oncogenic functions *via* sponging miR-183-5p in NPC. Furthermore, we explored whether glutamate ammonia ligase (GLUL) was a downstream target of miR-183-5p. Our results showed that OIP5-AS1 performed its biological functions *via* targeting miR-183-5p and GLUL in NPC cells.

## Materials and Methods

### Cell Culture and Reagents

Human NPC cells(CNE1, CNE2 and HNE1), which are Epstein-Barr virus (EBV)-negative cells, were cultured in RPMI-1640 medium supplemented with 10% fetal bovine serum (FBS). MTT [3-(4,5-dimethythi-azol-2-yl)-2,5-diphenyl tetrazolium bromide] was obtained from Sigma Company. Matrigel was bought from BD Biosciences Company. Lipofectamine 3000 was obtained from Invitrogen Company. The anti-GLUL and anti-tubulin antibodies were purchased from Cell Signaling Technology Company.

### Cell Viability Assay

The transfected CNE1 and CNE2 cells were seeded in 96-well plates for 72 hours. MTT assay was performed to measure cell viability by a spectrophotometer at 570 nm as described previously (25).

### Transfection

The NPC cells were seeded in 60 mm dishes overnight and then transfected with different plasmids (GenePharma, Shanghai, China) using Lipofectamine 3000 following the manufacture’s instruments (25, 26).

### Real-Time Quantitative RT-PCR

Total RNAs were extracted from the transfected NPC cells and then reversely transcribed into cDNA. The RT-1PCR was performed as described before (26). The mRNA levels were calculated using ^ΔΔ^Ct methods.

### Wound Healing Assay

The transfected NPC cells were seeded in 6-well plate. After the cells grew to around 100% confluence, the scratch wound was created using a pipette tip and the cells were washed by PBS. The cells were cultured for 20 hours. The photographs were taken at 0 hour and 20 hours, respectively.

### Transwell Matrigel Invasion Assay

The invasive activity of NPC cells was determined by Transwell Matrigel invasion assay using 24-well Transwell inserts with precoated Matrigel. The transfected NPC cells were seeded in upper level of the inserts with serum-free medium. The bottom level of the insert was added completed medium. After 24 hours, the cells that invaded on the bottom level were stained and photographed by a microscope.

### Western Blotting Analysis

The transfected cells were washed and harvested and then lysed by protein lysis buffer. The concentrations of proteins were measured using BCA assay. After SDS-PAGE was used to separate the protein samples, the proteins were transferred onto PVDF membranes. The membranes were incubated with 5% milk for 1 hour and then immunoblotted with anti-GLUL antibody overnight at cold room. Then, TBST was used to wash the membranes for three times and subsequently probed with the proper secondary antibody for 1 hour. The expression level of proteins was measured by ECL assay. Tubulin expression was used to act as a control.

### Dual Luciferase Reporter Gene Analysis

The GLUL wild-type, GLUL mutant, OIP5-AS1 wild-type, and OIP5-AS1 mutant were amplified and cloned in pmirGLO vector with luciferase. Cells were treated with different plasmids. OIP5-AS1 mutant has the mutated binding sites of miR-183-5p. GLUL mutant has the mutated binding sites of miR-183-5p. After 48 hours, the dual luciferase reporter gene analysis was detected following the manufacturer’s protocols (Promega, Madison, WI, USA).

### Statistical Analysis

Statistical analysis was measured by GraphPad Prism 5.0 (CA, USA). The significance was analyzed using the two-tailed Student’s *t-test* for comparing with two different groups. ANOVA was used for comparing with multiple groups. Data are shown with means ± SEM.

## Results

### Inhibition of OIP5-AS1 Suppressed Cell Viability in NPC Cells

**Figure 1:**
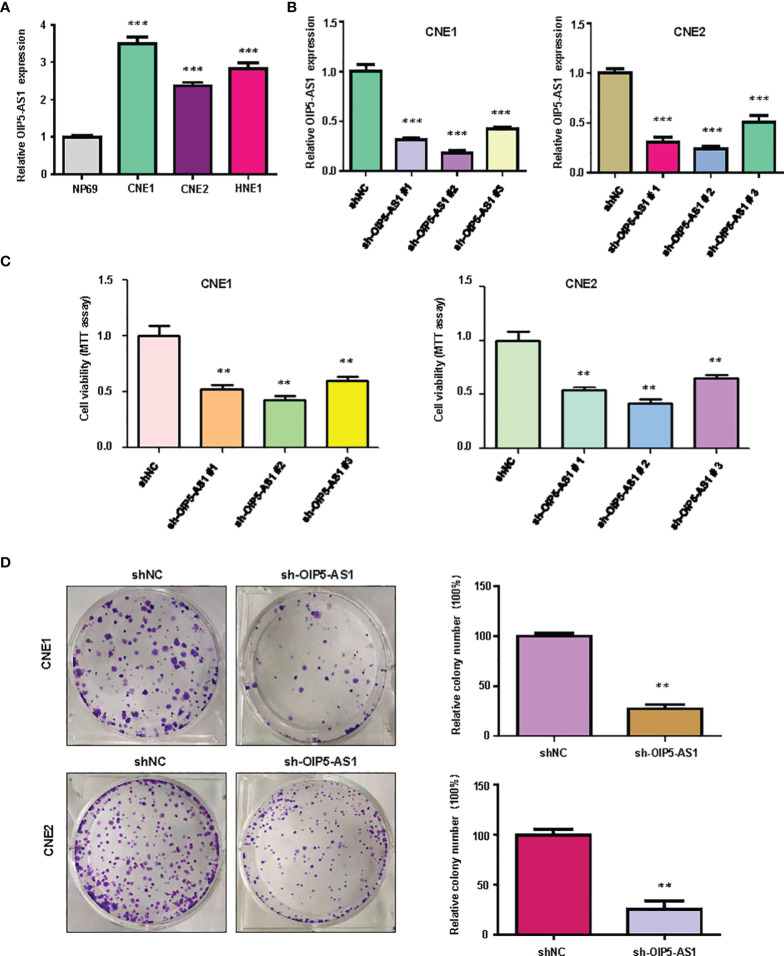
Knockdown of OIP5-AS1 inhibits viability of NPC cells. **(A),** RT-PCR assay was used to measure the expression of OIP5-AS1 in several NPC cell lines. ***P<0.001 vs control. **(B),** RT-PCR was used to detect the efficacy of OIP5-AS1 knockdown in CNE1 and CNE2 cells. ***P<0.001 vs control. **(C),** MTT assay was used to measure the viability of NPC cells after OIP5-AS1 knockdown. **P<0.01 vs control. **(D),** Cell colony formation was performed in NPC cells after OIP5-AS1 knockdown (Left panel). Quantitative data are represented (Right panel). **P<0.01 vs control.

We examined the expression of lncRNA OIP5-AS1 in NP69, CNE1, CNE2 and HNE1 cell lines by real-time RT-PCR analysis. We found that the expression of OIP5-AS1 was highly expressed in NPC cells compared with NP69 nasopharyngeal normal cell line (**Figure 1A**). Next, we used shRNA to knockdown the expression of OIP5-AS1 in CNE1 and CNE2 cells, which had the high expression of OIP5-AS1. We found that sh-OIP5-AS1 transfection led to downregulation of OIP5-AS1 in both CNE1 and CNE2 cells (**Figure 1B**). In the following study, we used sh-OIP5-AS1#2 to investigate its function in CNE1 and CNE2 cells. To measure the effect of OIP5-AS1 downregulation in NPC cells, we examined the cell viability in CNE1 and CNE2 cells after OIP5-AS1 knockdown. We observed that knockdown of OIP5-AS1 attenuated the viability of NPC cells (**Figure 1C**). Moreover, we performed the colony formation study to further determine the function of OIP5-AS1 in NPC cells. Our data showed that depletion of OIP5-AS1 reduced the colony formation activity in CNE1 and CNE2 cells (**Figure 1D**). Altogether, knockdown of OIP5-AS1 inhibited viability of NPC cells.

### Inhibition of OIP5-AS1 Reduced Migration and Invasion in NPC Cells

**Figure 2:**
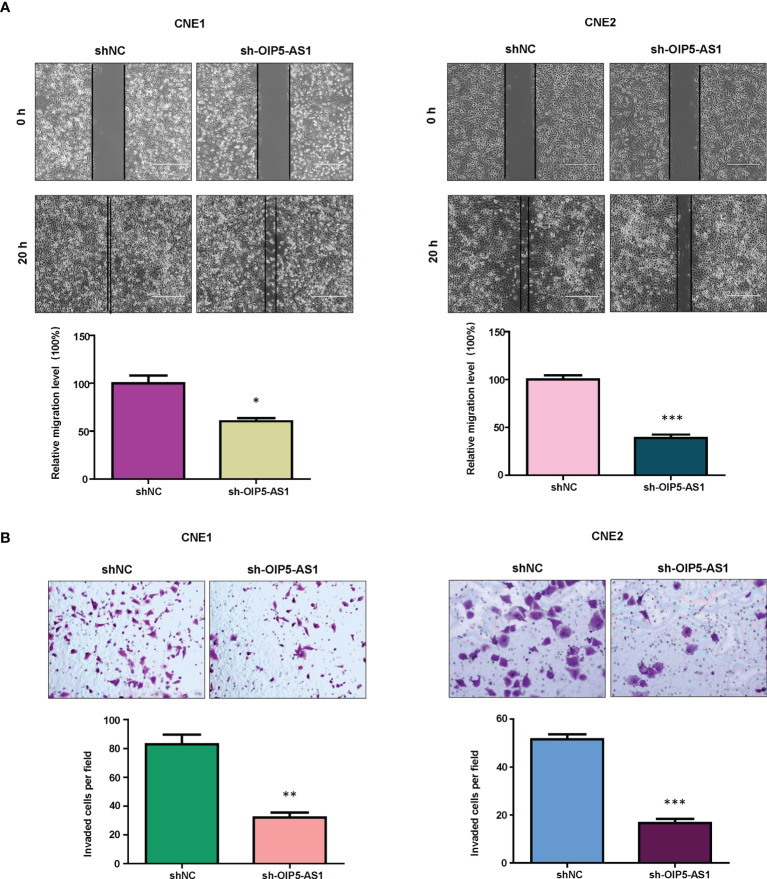
Knockdown of OIP5-AS1 inhibits migration and invasion of NPC cells. **(A),** Wound healing assays were utilized to measure the migratory activity of NPC cells after OIP5-AS1 knockdown (Top panel). Quantitative data are represented (Bottom panel). * P<0.05 vs control; ***P<0.001 vs control. **(B),** Transwell matrigel invasion analysis was utilized to test the invasiveness capacity of NPC cells after OIP5-AS1 knockdown (Top panel). Quantitative data are represented (Bottom panel) ** P<0.01 vs control; ***P<0.001 vs control.

It is known that OIP5-AS1 participates in cell migration and invasion in cancer. Therefore, we measured the migratory and invasive capacity of NPC cells after OIP5-AS1 knockdown. Our wound healing assay data showed that knockdown of OIP5-AS1 retarded would closure in both CNE1 and CNE2 cells (**Figure 2A**). Moreover, transwell invasion assay data demonstrated that knockdown of OIP5-AS1 reduced the invasiveness activity in CNE1 and CNE2 cells (**Figure 2B**). Taken together, OIP5-AS1 knockdown retarded the migrative and invasive capacity in NPC cells.

### OIP5-AS1 Targets miR-183-5p in NPC Cells

**Figure 3:**
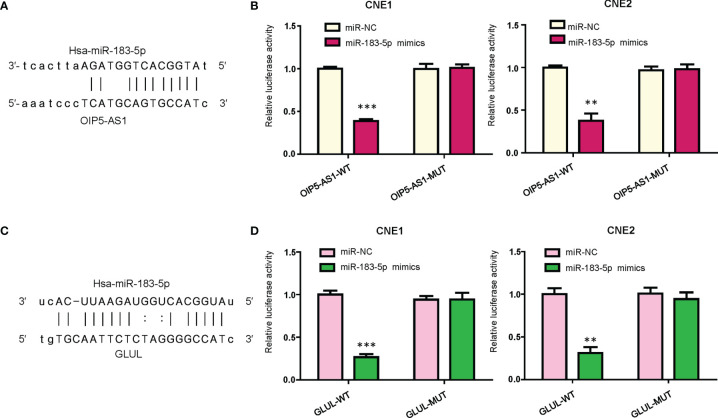
OIP5-AS1 targets miR-183-5p. **(A),** Potential binding sites between OIP5-AS1 and miR-183-5p are shown. **(B),** Dual luciferase reporter assays were used to verify the binding sites of miR-183-5p to OIP5-AS1. **P<0.01; ***P<0.001 vs control. **(C),** Potential binding sites between GLUL and miR-183-5p are shown. **(D),** Dual luciferase reporter assays were used to verify the binding sites of miR-183-5p to GLUL. **P<0.01; ***P<0.001 vs control.

According to the database from website RAID v2.0, OIP5-AS1 could bind to hsa-miR-183-5p. Moreover, the data from mircode.org also showed that OIP5-AS1 can interact with hsa-miR-183-5p. There were binding sites between miR-183-5p and lncRNA OIP5-AS1 (**Figure 3A**). To further validate this concept, we performed the dual luciferase reporter gene analysis. We found that the luciferase activity of OIP5-AS1 was decreased in the miR-183-5p mimics group when compared with miRNA control group (**Figure 3B**). In consistent, the activity of OIP5-AS1 mutation did not change in the both miR-183-5p mimics and miRNA control groups (**Figure 3B**). This result indicated that OIP5-AS1 might bind to miR-183-5p in NPC cells.

### GLUL Is a Potential Target of miR-183-5p

We used bioinformatic analysis to predict the downstream target of miR-183-5p. From the several public algorithms, such as TargetScan, miRanda, microRNA.org, PicTar, GLUL was revealed to be a target of miR-183-5p. The GLUL sequence has specific binding regions to interact with miR-183-5p (**Figure 3C**). To demonstrate whether miR-183-5p bound to GLUL, we conducted the dual luciferase reporter gene analysis. We observed that the luciferase activity of the GLUL was inhibited in the miR-183-5p mimic transfection group, whereas its luciferase activity did not change in the miR-183-5p mutation group (**Figure 3D**). Thus, GLUL might be a potential downstream target of miR-183-5p in NPC cells.

### OIP5-AS1 and miR-183-5p Target GLUL Expression

**Figure 4:**
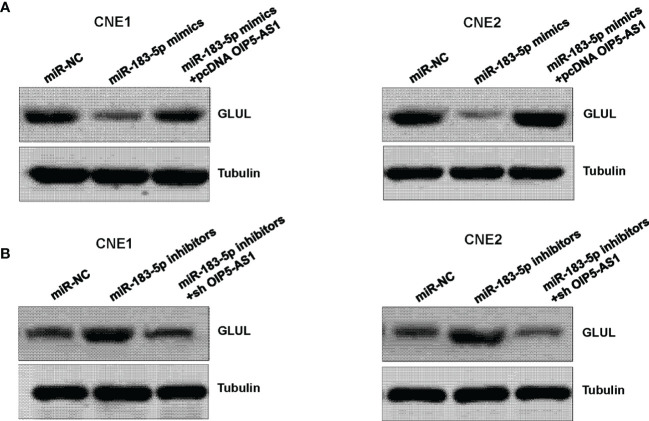
GLUL is a target of miR-183-5p. **(A),** Western blotting assay was performed to measure the expression of GLUL in NPC cells after miR-183-5p mimic and pcDNA OIP5-AS1 co-transfections. **(B),** Western blotting analysis was used to measure the expression of GLUL in NPC cells after miR-183-5p inhibitor and shRNA OIP5-AS1 co-transfections.

Next, we determined whether miR-183-5p could regulate the expression of GLUL in NPC cells. Our western blotting results showed that miR-183-5p mimic transfection inhibited the expression of GLUL in CNE1 and CNE2 cells (**Figure 4A**). Moreover, downregulation of miR-183-5p by its inhibitors increased the expression of GLUL in CNE1 and CNE2 cells (**Figure 4B**). Furthermore, overexpression of OIP5-AS1 rescued the inhibitory effect of miR-183-5p mimics on GLUL expression in NPC cells (**Figure 4A**). Consistently, depletion of OIP5-AS1 abrogated the promotive effect of miR-183-5p inhibitors on GLUL expression level in both NPC cell lines (**Figure 4B**). Therefore, GLUL might be a downstream factor of miR-183-5p.

### OIP5-AS1 Promotes Cell Viability *via* Regulating miR-183-5p in NPC Cells

**Figure 5:**
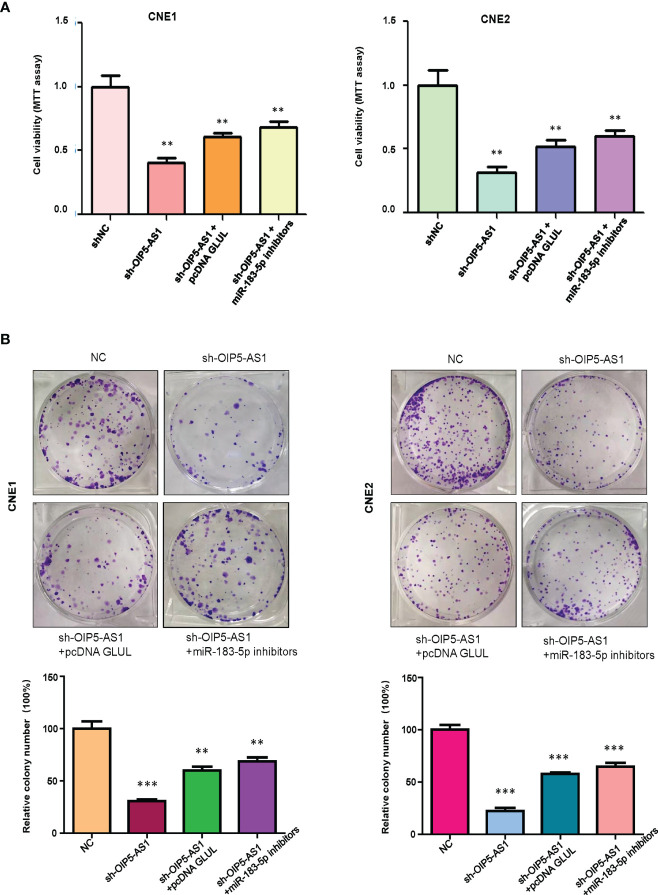
OIP5-AS1 knockdown suppressed cell viability *via* regulating miR-183-5p in NPC cells. **(A),** MTT assay was used to measure the viability of NPC cells after OIP5-AS1 knockdown, GLUL overexpression and miR-183-5p downregulation. **P<0.01 vs control. **(B),** Cell colony formation was performed in NPC cells after OIP5-AS1 knockdown, GLUL overexpression and miR-183-5p downregulation (Top panel). Quantitative data are represented (Bottom panel). **P<0.01; ***P<0.001 vs control.

We tested whether OIP5-AS1 governs cell viability *via* regulation of miR-183-5p in NPC cells. Our MTT assay showed that OIP5-AS1 downregulation inhibited cell viability in CNE1 and CNE2 cells (**Figure 5A**). Downregulation of miR-183-5p rescued the inhibitory effects of shRNA OIP5-AS1 transfection on cell viability in NPC cells (**Figure 5A**). Moreover, overexpression of GLUL abolished the suppression of cell viability by shRNA OIP5-AS1 transfection (**Figure 5A**). Consistently, colony formation experiments showed the similar trends in CNE1 and CNE2 cells (**Figure 5B**). Altogether, downregulation of OIP5-AS1 inhibited viability of NPC cells *via* regulation of miR-183-5p and its target GLUL.

### OIP5-AS1 Promotes Cell Motility *via* Regulating miR-183-5p and GLUL in NPC Cells

**Figure 6:**
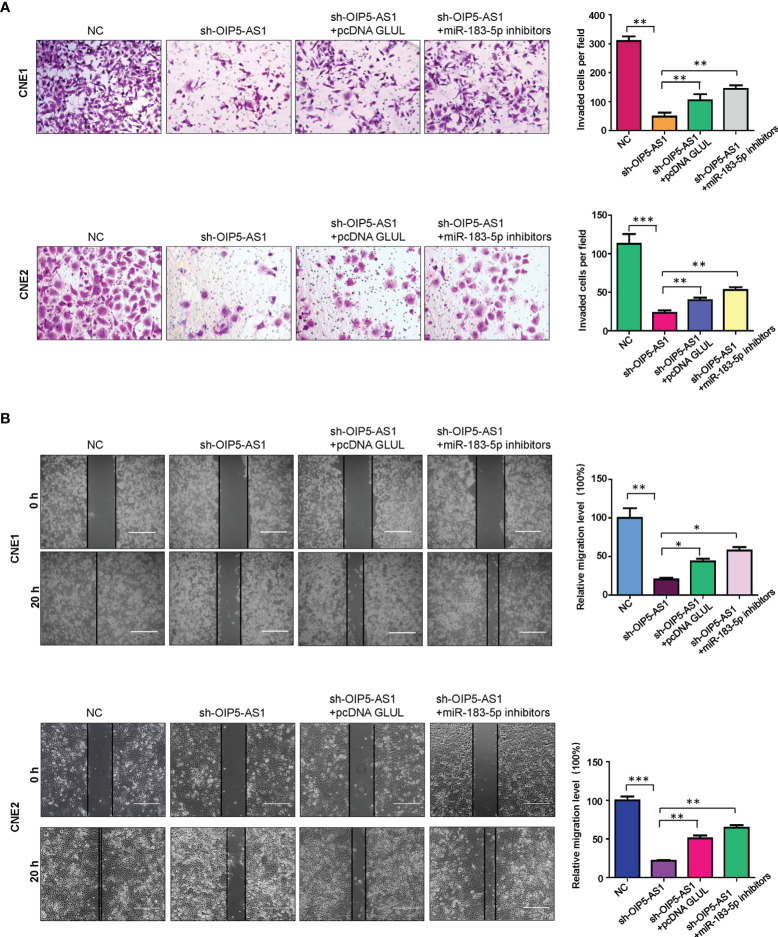
OIP5-AS1 knockdown inhibits cell motility *via* regulating miR-183-5p and GLUL in NPC cells. **(A),** Transwell Matrigel invasion analysis was utilized to test the invasiveness capacity of NPC cells after OIP5-AS1 knockdown, GLUL overexpression and miR-183-5p downregulation. (Left panel). Quantitative data are represented (Right panel) * P<0.05 vs control; ** P<0.01; ***P<0.001. **(B),** Wound healing assays were utilized to measure the migratory activity of NPC cells after OIP5-AS1 knockdown, GLUL overexpression and miR-183-5p downregulation (Left panel). Quantitative data are represented (Right panel). *P<0.05; **P<0.01; ***P<0.001.

We explored whether OIP5-AS1 regulates cell migration and invasion through targeting miR-183-5p and GLUL in NPC cells. The invasive ability was measured by Transwell invasion assay in NPC cells after modification of OIP5-AS1, GLUL and miR-183-5p. The results showed that shRNA OIP5-AS1 transfection reduced cell invasion capacity in CNE1 and CNE2 cells (**Figure 6A**). Overexpression of GLUL blocked the inhibitory function of OIP5-AS1 knockdown on cell invasion in NPC cells (**Figure 6A**). Moreover, suppression of miR-183-5p abolished the inhibitory effects on NPC cells that were induced by OIP5-AS1 knockdown (**Figure 6A**). Wound healing assay data demonstrated that knockdown of OIP5-AS1 retarded cell migratory ability, which could be rescued by overexpression of GLUL and inhibition of miR-183-5p in both NPC cell lines (**Figure 6B**). Taken together, OIP5-AS1 regulated cell invasion and migration *via* targeting miR-183-5p and GLUL in NPC cells.

## Discussion

LncRNAs have been reported to involve in carcinogenesis and tumor progression in many types of cancers (27–31). Evidence has revealed that OIP5-AS1 aggravated cell growth and migratory ability *via* interaction with EZH2 and downregulation of NLRP6 in gastric cancer (32). Wang et al. reported that OIP5-AS1 enhanced cell proliferation and promoted cell cycle *via* sponging miR-641 in gastric cancer (33). Song et al. observed that OIP5-AS1 facilitated cell proliferation and blocked apoptosis *via* targeting miR-143-3p/ROCK1 axis in cervical cancer (34). Tao et al. found that OIP5-AS1 increased cell growth and suppressed apoptosis *via* modulation of the miR-367-3p/HMGA2 pathway in gastric cancer (35). Moreover, OIP5-AS1 accelerated cell growth *via* affecting miR-422a and ANO1 axis in gastric cancer cells (36). Similarly, Zhi et al. found that OIP5-AS1 enhanced gastric cancer progression *via* sponging miR-153-3p and targeting ZBTB2 axis (37). Furthermore, OIP5-AS1 facilitated growth of pancreatic cancer cells *via* decoying miR-342-3p and activation of AKT/ERK pathway (38). OIP5-AS1 silencing led to inhibition of cell proliferation and apoptosis in 5-8F cells and CNE1 cells *via* sponging miR-203 in NPC (24). Herein, our study showed that OIP5-AS1 downregulation inhibited the viability of NPC *via* targeting miR-183-5p.

OIP5-AS1 was reported to enhance the proliferation, motility activity and EMT *via* decoying miR-204-5p and increasing ZEB1 in laryngeal squamous cell carcinoma (39). Knockdown of OIP5-AS1 repressed proliferation and migration *via* regulating miR-3163/VEGFA axis in liver cancer cells (40). Moreover, OIP5-AS1 facilitated the tumor malignant progression *via* targeting miR-429/FOXD1/ERK axis in pancreatic cancer (41). OIP5-AS1 silencing led to inhibition of cell migration and invasion in 5-8F and CNE1 cells *via* sponging miR-203 in NPC (24). Our study demonstrated that OIP5-AS1 knockdown suppressed cell invasion and migration *via* regulating miR-183-5p and GLUL in NPC cells. Tang et al. found that miR-183-5p is a biomarker for patients with NPC (42). This study reported that miR-183-5p expression was negatively associated with lymph node status in NPC patients (42). GLUL has been identified to participate in carcinogenesis and tumor progression (43). GLUL is involved in tumorigenesis in a variety of cancers; however, the function of GLUL in NPC is unclear. Here, we found that GLUL was involved in OIP5-AS1-mediated tumor promotion in NPC cells.

It has been reported that OIP5-AS1 is involved in drug resistance in various malignancies. Song et al. reported that OIP5-AS1 increased cisplatin resistance *via* binding with miR-340-5p and upregulating LPAATβ and PI3K/AKT/mTOR pathway in osteosarcoma (44). Similarly, OIP5-AS1 promoted cisplatin resistance *via* regulating miR-27b-3p/TRIM14 axis in oral squamous cell carcinoma (45). Liang et al. reported that OIP5-AS1 targeted miR-137 and increased L-OHP sensitivity in colon cancer cells (46). Another group found that OIP5-AS1 increased doxorubicin resistance *via* sponging miR-137-3p and upregulating PTN in osteosarcoma (47). Moreover, exosomal-OIP5-AS1 promoted trastuzumab chemoresistance *via* decoying miR-381-3p and increasing HMGB3 in breast cancer (48). OIP5-AS1 increased cell resistance to imatinib *via* targeting miR-30e-5p and ATG12 in chronic myeloid leukemia cells (49). However, it is unclear whether OIP5-AS1 is involved in drug resistance in NPC, which needs to further investigate.

## Conclusion

In summary, OIP5-AS1 downregulation suppressed the viability, migration and invasion of NPC *via* targeting miR-183-5p. GLUL might be a potential downstream target of miR-183-5p in NPC cells. Moreover, OIP5-AS1 promotes cell motility *via* regulating miR-183-5p and GLUL in NPC cells. Therefore, OIP5-AS1 exerted its biological functions *via* targeting miR-183-5p and GLUL in NPC.

## Data Availability Statement

The original contributions presented in the study are included in the article/**Supplementary Material**. Further inquiries can be directed to the corresponding authors.

## Author Contributions

SL performed experiments and wrote the manuscript, MT performed data analysis, NZ, JL, XX, DH, and FL conceived and designed the study, revised the manuscript. XZ supervised the study and edited the manuscript. All authors have approved the final manuscript.

## Funding

This study was supported by grants from the Science and Technology Foundation of Shenzhen (JCYJ20180302144624391, JCYJ20210324112607020), and the Science and Technology Foundation of Nanshan District (2020112).

## Conflict of Interest

The authors declare that the research was conducted in the absence of any commercial or financial relationships that could be construed as a potential conflict of interest.

## Publisher’s Note

All claims expressed in this article are solely those of the authors and do not necessarily represent those of their affiliated organizations, or those of the publisher, the editors and the reviewers. Any product that may be evaluated in this article, or claim that may be made by its manufacturer, is not guaranteed or endorsed by the publisher.
