# Supplementary material for: LncRNA OIP5-AS1 Knockdown Targets miR-183-5p/GLUL Axis and Inhibits Cell Proliferation, Migration and Metastasis in Nasopharyngeal Carcinoma
Source: Front Oncol. 2022 Jun 8;12:921929. doi: 10.3389/fonc.2022.921929 (PMC9214031; doi:10.3389/fonc.2022.921929)
Supplement: Supplementary file 1 [file DataSheet_1.pdf]

F1g1

Relative OIP5-AS1 expression			
HOB	CNE1	CNE2	HNE1
1.05	3.82	2.12	2.83
1.02	3.21	2.43	2.56
0.93	3.45	2.45	3.11

CNE1-Group	Relative OIP5-AS1 expression
shNC	1.01
	0.88
	1.12
sh-OIP5-AS1 #1	0.33
	0.28
	0.34
sh-OIP5-AS1 #2	0.12
	0.22
	0.2
sh-OIP5-AS1 #3	0.44
	0.39
	0.45

CNE2-Group	Relative OIP5-AS1
shNC	1.03
	0.93
	1.07
sh-OIP5-AS1 #1	0.41
	0.28
	0.25
sh-OIP5-AS1 #2	0.22
	0.33
	0.21
sh-OIP5-AS1 #3	0.44
	0.64
	0.45

CNE1-Group	Colony Count	Mean (control)	(fold) /control
shRNA	205	205	100
	215		105
	195		95
sh-OIP5-AS1	57		28
	41		20
	70		34

CNE2-Group	Colony Count	Mean (control)	(fold) /control
shRNA	329	329	100
	296		90
	362		110
sh-OIP5-AS1	82		25
	132		40
	39		12
