# Supplementary material for: LncRNA OIP5-AS1 Knockdown Targets miR-183-5p/GLUL Axis and Inhibits Cell Proliferation, Migration and Metastasis in Nasopharyngeal Carcinoma
Source: Front Oncol. 2022 Jun 8;12:921929. doi: 10.3389/fonc.2022.921929 (PMC9214031; doi:10.3389/fonc.2022.921929)
Supplement: Supplementary file 2 [file DataSheet_2.pdf]

F1g2

wound healing assay

CNE1-Group	0h Area	20h Area	20h rate	Fold change 20h TEST/NC
shRNA	77.11%	98.63%	94.01%	1.014568699
	79.70%	96.90%	84.73%	0.914360369
	75.98%	99.82%	99.25%	1.071070932
sh-OIP5-AS1	79.38%	90.46%	53.73%	0.579877269
	80.32%	92.47%	61.74%	0.666248385
	76.39%	88.49%	51.25%	0.553062699

wound healing assay

CNE2-Group	0h Area	20h Area	20h rate	Fold change 20h TEST/NC
shRNA	76.89%	94.67%	76.94%	0.999912766
	78.05%	93.64%	71.03%	0.923085422
	76.01%	95.89%	82.87%	1.077001812
sh-OIP5-AS1	78.39%	84.07%	26.28%	0.341604726
	76.54%	85.27%	37.21%	0.483633681
	76.78%	82.79%	25.88%	0.336389598

CNE1-Group	invasive cells		CNE2-Group	invasive cells
shRNA	80		shRNA	52
	96			48
	73			55
sh-HOXA-AS3	38		sh-HOXA-AS3	14
	26			20
	32			16
