# Supplementary material for: LncRNA OIP5-AS1 Knockdown Targets miR-183-5p/GLUL Axis and Inhibits Cell Proliferation, Migration and Metastasis in Nasopharyngeal Carcinoma
Source: Front Oncol. 2022 Jun 8;12:921929. doi: 10.3389/fonc.2022.921929 (PMC9214031; doi:10.3389/fonc.2022.921929)
Supplement: Supplementary file 3 [file DataSheet_3.pdf]

F1g3

Relative OIP5-AS1 expression	Ago2	Ago2	Ago2	IgG	IgG	IgG
miR-NC	2.81	2.56	2.23	6.78	7.21	6.56
miR-183-5P mimics	1.12	0.98	0.92	1.22	1.01	0.95

Relative OIP5-AS1 enrichment			
NC-Bio	1.21	24.26	4.08
miR-183-5p-Bio	0.82	28.03	6.19
miR-183-5p-Bio-MUT	0.97	20.1	7.96

CNE1-Group Relative luciferase	miR-NC	miR-NC	miR-NC	miR-183-5	miR-183-5	miR-183-5p-Bio
OIP5-AS1 -WT	1.00	0.96	1.04	0.35	0.43	0.38
OIP5-AS1 -MUT	1.09	1.02	0.88	0.98	0.96	1.09

CNE2-Group Relative luciferase	miR-NC	miR-NC	miR-NC	miR-183-5	miR-183-5	miR-183-5p-Bio
OIP5-AS1 -WT	1.02	0.94	1.04	0.26	0.33	0.54
OIP5-AS1 -MUT	1.02	1.00	0.88	0.89	0.96	1.09

CNE1-Group Relative luciferase	miR-NC	miR-NC	miR-NC	miR-183-5	miR-183-5	miR-183-5p-Bio
GLUL-WT	1.00	0.92	1.08	0.26	0.33	0.21
GLUL-MUT	0.92	1.02	0.88	0.80	0.96	1.07

CNE2-Group Relative luciferase	miR-NC	miR-NC	miR-NC	miR-183-5	miR-183-5	miR-183-5p-Bio
GLUL-WT	0.98	0.89	1.13	0.19	0.33	0.42
GLUL-MUT	1.12	1.02	0.88	0.80	0.96	1.07
