# Supplementary material for: LncRNA OIP5-AS1 Knockdown Targets miR-183-5p/GLUL Axis and Inhibits Cell Proliferation, Migration and Metastasis in Nasopharyngeal Carcinoma
Source: Front Oncol. 2022 Jun 8;12:921929. doi: 10.3389/fonc.2022.921929 (PMC9214031; doi:10.3389/fonc.2022.921929)
Supplement: Supplementary file 5 [file DataSheet_5.pdf]

Fig 5

CNE1-Group	Colony Count	Mean (control)	(fold) /control
NC	192	179	107
	190		105
	154		86
sh-OIP5-AS1	54		30
	50		28
	61		34
sh-OIP5-AS1 +pcDNA GLUL	120		67
	104		58
	98		55
sh-OIP5-AS1+miR-183-5P inhibitors	115		64
	136		76
	118		66

cne2-Group	Colony Count	Mean (control)	(fold) /control
NC	316	310	102
	332		107
	282		91
sh-OIP5-AS1	65		21
	87		28
	56		18
sh-OIP5-AS1 +pcDNA GLUL	171		55
	180		58
	186		60
sh-OIP5-AS1+miR-183-5P inhibitors	217		70
	180		58
	205		66
