# Supplementary material for: LncRNA OIP5-AS1 Knockdown Targets miR-183-5p/GLUL Axis and Inhibits Cell Proliferation, Migration and Metastasis in Nasopharyngeal Carcinoma
Source: Front Oncol. 2022 Jun 8;12:921929. doi: 10.3389/fonc.2022.921929 (PMC9214031; doi:10.3389/fonc.2022.921929)
Supplement: Supplementary file 6 [file DataSheet_6.pdf]

	A	B	C	D	E	F
1	F1g 6					
2	CNE1-Group	Invasive cells		CNE2-Group	invasive cells	
3	NC	300		NC	114	
4		288			134	
5		340			90	
6	sh-OIP5-AS1	76		sh-OIP5-AS1	23	
7		38			18	
8		30			29	
9		79			46	
10	sh-OIP5-AS1	147		sh-OIP5-AS1	35	
11	+pcDNA GLUL	88		+pcDNA GLUL	38	
12		128			50	
13	sh-OIP5-AS1+miR-183-5P inhibitors	167		sh-OIP5-AS1+miR-18	60	
14		138			49	
15						

16						
17	wound healing assay					
18	CNE1-Group	0h Area	20h Area	20h rate	Fold change 20h TEST/NC	
19	NC	72.36%	98.78%	95.59%	119.19%	
20		73.89%	93.89%	76.60%	95.52%	
21		75.35%	92.21%	68.40%	85.29%	
22	sh-OIP5-AS1	73.26%	78.26%	18.70%	23.32%	
23		70.01%	74.27%	14.20%	17.71%	
24		71.08%	76.18%	17.63%	21.99%	
25		71.02%	82.56%	39.82%	49.66%	
26	sh-OIP5-AS1	72.38%	80.50%	29.40%	36.66%	
27	+pcDNA GLUL	73.09%	82.73%	35.82%	44.67%	
28		71.25%	82.56%	39.34%	49.05%	
29	sh-OIP5-AS1+miR-183-5P inhibitors	72.08%	85.25%	47.17%	58.82%	
30		71.20%	86.31%	52.47%	65.42%	
31						

32						
33	wound healing assay					
34	CNE2 Group	0h Area	20h Area	20h rate	Fold change 20h TEST/NC	
35	NC	75.42%	94.48%	77.54%	97.23%	
36		73.89%	96.26%	85.68%	107.43%	
37		75.72%	94.18%	76.03%	95.34%	
38	sh-OIP5-AS1	76.32%	80.22%	16.47%	20.65%	
39		74.28%	79.02%	18.43%	23.11%	
40		75.53%	79.39%	15.77%	19.78%	
41		75.29%	84.32%	36.54%	45.82%	
42	sh-OIP5-AS1	76.38%	87.27%	46.10%	57.81%	
43	+pcDNA GLUL	74.97%	84.72%	38.95%	48.84%	
44		75.45%	87.03%	47.17%	59.15%	
45	sh-OIP5-AS1+miR-183-5P inhibitors	78.11%	90.28%	55.60%	69.71%	
46		77.04%	87.87%	47.17%	59.15%	
47						
